# Perceived stress and resilience and their relationship with the use of mobile phone among nursing students

**DOI:** 10.17533/udea.iee.v37n3e05

**Published:** 2019-10-23

**Authors:** Maya Sahu, Sailaxmi Gandhi, Manoj Kumar Sharma, P. Marimuthu

**Affiliations:** 1 RN, RM, BSN, MSN. Ph.D Scholar, Department of Nursing, National Institute of Mental Health & Neurosciences (Institute of National Importance), India. Email: mayamonsahu@gmail.com National Institute Of Mental Health And Neurosciences National Institute of Mental Health & Neurosciences (Institute of National Importance) India mayamonsahu@gmail.com; 2 RN, RM, BSN, MSN, Ph.D, Additional Profesor & Head, Department of Nursing, National Institute of Mental Health & Neurosciences (Institute of National Importance) India. Email: sailaxmi63@yahoo.com. Corresponding author National Institute Of Mental Health And Neurosciences National Institute of Mental Health & Neurosciences (Institute of National Importance) India sailaxmi63@yahoo.com; 3 M.Phil (Medical & Social Psychology), Ph.D, Profesor, Department of Clinical Psychology, National Institute of Mental Health & Neurosciences (Institute of National Importance) India. Email: shutclinic@gmail.com National Institute Of Mental Health And Neurosciences National Institute of Mental Health & Neurosciences (Institute of National Importance) India shutclinic@gmail.com; 4 . BSc (Statistics), MSc (Statistics), PhD (Science), Department of Biostatistics, National Institute of Mental Health & Neurosciences (Institute of National Importance) India. Email: p_marimuthu@hotmail.com National Institute Of Mental Health And Neurosciences National Institute of Mental Health & Neurosciences (Institute of National Importance) India p_marimuthu@hotmail.com

**Keywords:** physiological, resilience, psychological, students, nursing, cell phone use, cross-sectional studies, surveys and questionnaires., estrés fisiológico, resiliencia psicológica, estudiantes de enfermería, uso del teléfono celular, estudios transversales, encuestas y cuestionarios., estresse fisiológico, resiliência psicológica, estudantes de enfermagem, uso do telefone celular, estudos transversais, inquéritos e questionários.

## Abstract

**Objective.:**

The study sought to explore the relationship between levels of stress and resilience with the use of the mobile phone in nursing students.

**Methods.:**

Cross-sectional study conducted with 102 nursing students from several Nursing schools in India who were invited to participate in the research. The data were gathered by using the following instruments: Perceived Stress Scale (PSS) by Cohen, The Connor-Davidson Resilience scale (CD-RISC), and Mobile Phone Involvement Questionnaire (MPIQ) by Walsh.

**Results.:**

Most of the participants were women (94.1%), studying in the undergraduate (70.6%), with a mean age of 25.2 years. In all, 77.5% of the students had stress perception between moderate and high, 20.6% had high resilience capacity, and 25.5% were frequent mobile phone users. Perceived stress was correlated significantly and negatively with age and resilience capacity. Graduate students had greater capacity to recover than undergraduate students.

**Conclusion.:**

This study indicates the negative relation of resilience capacity with stress and the use of mobile phones among nursing students. Hence, it is necessary for institutions preparing nurses to develop intervention strategies to enhance the resilience capacity, improve skills to manage stress, and healthy use of the mobile phone.

## Resumen

**Objetivo.:**

Explorar la relación entre los niveles de estrés y resiliencia con el uso del teléfono móvil en estudiantes hindúes de enfermería.

**Métodos.:**

Estudio de corte transversal realizado con 102 estudiantes de enfermería de varias escuelas de enfermería en la India a quienes se invitó a participar en la investigación. Los datos se recopilaron con la ayuda de los siguientes instrumentos: *Percibed Stress Scale* (PSS) de Cohen, *The Connor-Davidson Resilience scale* (CD-RISC) y *Mobile Phone Involvement Questionnaire* (MPIQ) *de* Walsh.

**Resultados.:**

La mayoría de los participantes fueron mujeres (94.1%), estudiaban pregrado (70.6%) y tenían un promedio de edad de 25.2 años. El 77.5% de los estudiantes tenía una percepción de estrés entre moderada y alta; el 20.6% tenía alta capacidad de resiliencia y el 25.5% eran usuarios frecuentes de teléfonos móviles. El estrés percibido se correlacionó significativamente y de manera negativa con la edad y la capacidad de resiliencia. Los estudiantes de posgrado tuvieron mayor capacidad de recuperación que los estudiantes de pregrado.

**Conclusión.:**

El presente estudio indica la relación negativa de la capacidad de resiliencia con el estrés y el uso de teléfonos móviles entre los estudiantes hindúes de enfermería. Por lo tanto, es necesario que las instituciones formadoras de enfermeros desarrollen estrategias de intervención para fortalecer la capacidad de resiliencia, mejorando las habilidades para manejar el estrés y el uso saludable del teléfono móvil.

## Resumo

**Objetivo.:**

Explorar a relação entre os níveis de estresse e resiliência com o uso do telefone móbil em estudantes de enfermagem.

**Métodos.:**

Estudo de corte transversal realizado com 102 estudantes de enfermagem de várias escolas de enfermagem na Índia que foram convidados a participar na investigação. Os dados se recopilaram utilizando os seguintes instrumentos: *Percibed Stress Scale* (PSS) de Cohen, *The Connor-Davidson Resilience scale* (CD-RISC) e *Mobile Phone Involvement Questionnaire* (MPIQ) *de* Walsh.

**Resultados.:**

A maioria dos participantes eram mulheres (94.1%), estudavam graduação (70.6%)e tinham uma média de idade de 25.2 anos. 77.5% dos estudantes tinham uma percepção de estresse entre moderada e alta, 20.6% tinha alta capacidade de resiliência e 25.5% eram usuários frequentes de telefones móbeis. O estresse percebido se correlacionou significativamente e de maneira negativa com a idade e a capacidade de resiliência. Os estudantes de pós-graduação tiveram maior capacidade de recuperação que os estudantes de graduação.

**Conclusão.:**

O presente estudo indica a relação negativa da capacidade de resiliência com o estresse e o uso de telefones móbeis entre os estudantes de enfermagem. Por tanto, é necessário que as instituições formadoras de enfermeiros desenvolvem estratégias de intervenção para fortalecer a capacidade de resiliência, melhorando as habilidades para manejar o estresse e o uso saudável do telefone móbil.

## Introduction

Stress is an emerging issue in the present life. There are many factors that lead to stress among the nursing students. The effect of perceived stress on students’ health depends on their coping abilities and resilience as well. Stress and mobile phone use has been found to have bi-directional interaction. For example, mobile phone can be used by some students as stress relieving gadget and similarly, students who use mobile phone excessively may be stressed because of constant connection with others. Psychological stress among the students especially those who are pursuing a professional degree, is one of the major factors that affect students’ academic performance and satisfaction.(1) Research focusing on nurses has commonly found that this population reports high levels of stress.(2) Stress has been defined as ‘a strain that accompanies a demand perceived to be either challenging (positive) or threatening (negative) and depending on the appraisal either adaptive or debilitating’.(3) Lazarus & Folkman in 1984 defined stress as a ‘Particular relationship between the person and the environment that is appraised by the person as taxing or exceeding his or her resources and endangering his or her wellbeing’.(4) Stress is perceived when an individual feels an inability in managing the stressors either due to inadequate abilities or resources.(5) Previous literature has found perceived stress to be associated with self-efficacy, workload,(6) poorer physical, psychological, and social health.(1)

The sources of stressors reported among nursing students are: academic, clinical and personal sources.(7,8) Along with other personal problems, financial concerns and lack of free time play major role as stressors.(7) Work/life imbalance is a significant stressor for female nursing students with children.(9) The effect of perceived stress on students’ health depends on their coping abilities. Resilience is a coping ability to keep on trying and bouncing back on adverse circumstances.(10) Resilience is related to positive emotions and these positive emotions during stressful experience have adaptive benefits in coping with stress.(11) Thus, the individual with good resilience tends to perceive less stress in a difficult environment and overcome the stressful situation easier than the one with less resilience. 

High mobile phone use is associated with stress, sleep disturbances and symptoms of depression for the young adults,(12) whereas a significant cross-sectional association was found between problematic mobile phone use, psychological distress and academic performance among college students.(13) High mobile phone use can lead to stress, depression and anxiety or vice-versa. The studies mentioned so far have tried to identify the relationship of stress and resilience either individually or combined with other factors affecting mental health. Further, few studies have assessed the impact of mobile phone use on stress, sleep, self-esteem and depression.(12,13) But the present study is one of its kind which assesses the level of stress and resilience and their relationship with use of mobile phone among the nursing students.

The objectives of the study are 1) To explore level of stress, resilience and mobile phone use among nursing students. 2) To find out the relationship between those variables. 3) To find out association between the level of stress, resilience and mobile phone use with socio demographic variables, such as gender and education. The study also sought for implications for the academicians regarding the same.

## Methods

This is a cross-sectional descriptive study based on convenience sampling. Undergraduate and postgraduate nursing students from various colleges of nursing in India who came for clinical experience were invited to participate in the study.

### Sample.

In 2017 when the study was being conducted, students from total 15 colleges had come to the said institute for clinical experience and out of them, 9 colleges were willing to take part in the study. All possible sample was considered for the study. However, post-hoc analysis for sample size calculation was done with known population mean and SD from previous study.(14) Fixing alpha at 0.05 and power at 80%, the sample size was estimated to be minimum 98. Hence, the questionnaires were distributed to 110 students (postgraduates=32 and undergraduates=78). However, 8 uncompleted questionnaires were discarded and the final sample comprised of 102 with 92.7% response rate.

### Procedure.

The study was approved by the Institute Ethics Committee. Participants were explained briefly about the study and asked if they would consider participating. Permission was obtained from the respective college Principals or teachers. Consenting participants were informed in details about the assessments. Informed consent was obtained from the participants. Both male and female students were included. Students who used substances in any form were excluded from the study. The details of current/lifetime use of any substance were elicited in the demographic data sheet. It helped in excluding students with substance use. It was an attempt to address the confounding variable in the current study on assessing the psychological correlates of mobile phone use. However, none of the participants had a history of current/lifetime use of any substance use.

### Measures.

4 types of information sources were used: *1)* S*ocio-demographic data sheet* was used to find out the age, gender and education of the study participants; *2) Perceived stress scale -PSS-* (15) is one of the most commonly used tools across different countries, cultures and population characteristics. It aims at accessing the degree to which situational changes in one’s life are perceived as stressful. The 10-items version was used in this study. It has a good internal consistency with alpha coefficient of 0.85. Individual scores on the PSS can range from 0 to 40 with higher scores indicating higher perceived stress. Scores ranging from 0-13 is considered low stress, scores ranging from 14-26 as moderate stress and scores ranging from 27-40 as high perceived stress; *3) Connor-Davidson Resilience Scale (CD-RISC):*(16) The CD-RISC is a 25-item scale that measures the ability to cope with stress and adversity. Studies reported the internal consistency for the CD-RISC scale as 0.89 and 0.92, respectively.(16,17) Based on quartile deviation, resilience is scored for this study as ≤ 61 low or less resilience, 62-79 moderate resilience and ≥ 80 high or good resilience; and *4) Mobile Phone Involvement Questionnaire (MPIQ;)*(18) MPIQ is an 8-item measure of mobile phone involvement on a 7-point Likert scale with good construct validity. Cronbach’s alpha shows acceptable reliability (α=0.81) for this questionnaire. Based on quartile deviation in this study, mobile phone use is considered low when score is ≤20, moderate if scores range from 20-35 and high when score is ≥36.

### Statistical analysis.

Data were analyzed using SPSS-22. Descriptive statistics such as frequency and percentage were used to describe the categorical variables and mean and SD were identified for the continuous variables. Rrelationship between the perceived stress, resilience and use of mobile phone was identified by using Pearson’s correlation test and Chi-square was used for association between selected socio-demographic variables and the study variables.

## Results

Majority of the 102 students were female (94.1%) and undergraduate (70.6%). Mean age of the participants was 25.3±4.8 years. About the test: resilience was higher (71±11.5), the perceived stress was moderate (18.6±5.9) and mobile phone use was moderate too (27.4±8.6).

Eighty percent students had moderate to high perceived stress, 20.6% had high resilience and 25.5% were frequent mobile phone users (Table 1).

**Table 1:** Distribution of subjects based on level of perceived stress, resilience and mobile phone use

Level Test	Low %	Moderate % H	High %
Stress	22.5	67.7	9.8
Resilience	19.6	59.8	20.6
Mobile phone use	21.6	52.9	25.5

Table 2 shows that perceived stress was significantly negatively correlated with resilience (*r*=-0.196) and age (*r*=-0.295) and positively correlated with mobile phone use (*r*=0.216). Resilience was found to be significantly higher among the post graduate students than the undergraduates (F=10.4, *p*=0.002). No other study variable had significant association with socio demographic variables.

**Table 2:** Correlation between the variables of the research

Variables	MPIQ	PSS	Resilience
Age	-0.072	-0.295**	0.181
MPIQ		0.216*	-0.037
PSS			-0.196*

*p<0.05, **p<0.01

## Discussion

This study provides the preliminary findings on the level of perceived stress, resilience and their relationship with the usage of mobile phone among nursing students. The present findings are corroborated with available literature among nursing profession. Nursing students are vulnerable to perceive more stress than other group of students. To be specific, they experience longer hours of study and associated lack of free time.(7) Studies documented moderate to high perceived stress among nursing students.(11,19) Eighty seven percent of the nursing students reported to have endured higher to much higher than average level of perceived stress.(1) Similar to the present study findings, the mean stress score was found to be 16.7±4.8 in a college student group which is little lower than the present study findings.(14) This emphasizes the fact that the nursing students experience more stress than the general college students. On the contrary, mean perceived stress score was seen to be 28.7±5.3 among nursing undergraduate students (*n*=282) in another study, which is alarming.(20) This discrepancy could be due to the use of different measures of perceived stress over studies. 

The current findings that resilience is significantly negatively correlated with perceived stress is in line with previous studies from other parts of the country or from outside India. A significant negative relationship (*r*=-0.7) has been seen between perceived stress and resilience of the respondents.(11) Resilience score was significantly found to be negatively correlated (*r*=-0.31) with perceived stress.(1)

In this study, perceived stress is negatively correlated with age and resilience was found to be significantly higher among the post graduate students than the undergraduates (F=10.4). It could be attributed to the fact that higher the level of maturity, higher the resilience and higher the resilience, lower the perception of stress. This trend is in accord with previous research showing that resilience increases with life experience and age.(21) High use of mobile phone is positively correlated with perceived stress. Prior literature has also shown that high use of mobile phone is associated with high level of stress. Similar to the present study findings, one study found that there were cross-sectional associations between high mobile phone use and stress, sleep disturbances, and symptoms of depression among the young adults as well.(12) Significant cross-sectional association was also reported between problematic mobile phone use and psychological distress, lower self-esteem, gender, smartphone use, multiple chatting applications, committed relationship status and frequency of mobile phone use.(13) 

### Strength and limitation.

The present study included students who came from many other states of India which may be representative of the country. Use of internationally standardized scale in the study and good response rate (92.7%) were main strength of the study. The study has also certain limitations such as cross sectional descriptive study, small sample size and the data was collected using self-reported questionnaires. As this study was conducted only with Indian nursing students, generalization of the findings in other countries has to be cautiously considered. 

### Recommendations.

The preliminary study findings show that there is higher perceived stress among the group which is negatively correlated with resilience. Stress is also found to be related with the use of mobile phone. This initial trend shows that there are implications of stress on use of mobile phone where resilience can be considered as a protective factor, but the correlation is low that needs to be validated through large sample size. Hence the implications for the study are to raise awareness of mental health issues. These findings highlight the importance of stress management and life skill training program and promotion of healthy use of technology in the colleges or universities, where the students can avail themselves with the abilities of managing stress or coming back easily from adverse situations and where the mobile phone will be used only when necessary, not as a stress relieving gadget.

## Conclusion.

The present study indicates the negative relationship of resilience with stress and mobile phone use among the nursing students. Thus, the study highlights the need for developing psycho-educational module or brief-intervention to strengthen their resilience, enhancing the skills to manage stress as well healthy use of mobile phone. The study also paves the way for academicians and administrators of colleges of nursing or other institutes for providing facilities and opportunities to the students for managing their stress which in turn equip them for better patient care.

## References

[1] Tung KS, Ning WW, Kris LTYA. (2014). Effect of Resilience on Self-Perceived Stress and Experiences on Stress Symptoms a Surveillance Report. Univers. J. Public Health..

[2] Galvin J, Suominen E, Morgan C, O’Connell E-J, Smith AP. (2015). Mental health nursing students’ experiences of stress during training: a thematic analysis of qualitative interviews. J. Psychiatr. Ment. Health Nurs..

[3] Sanders A, Lushington K. (2002). Effect of perceived stress on student performance in dental school. J. Dent. Educ..

[4] Folkman S. (2013). Stress: Appraisal and Coping. In: Gellman MD, Turner JR. (eds). Encyclopedia of Behavioral Medicine..

[5] Sood S, Bakhshi A, Devi P. (2013). An Assessment of Perceived Stress, Resilience and Mental Health of Adolescents Living in Border Areas. Int. J. Sci. Publication..

[6] Al-Sowygh ZH. (2013). Academic distress, perceived stress and coping strategies among dental students in Saudi Arabia. Saudi Dent. J..

[7] Gibbons C, Dempster M, Moutray M. (2008). Stress and eustress in nursing students. J. Adv. Nurs..

[8] Gibbons C, Dempster M, Moutray M. (2011). Stress, coping and satisfaction in nursing students. J. Adv. Nurs..

[9] Pryjmachuk S, Richards DA. (2007). Predicting stress in pre-registration nursing students. Br. J. Health Psychol..

[10] Earvolino‐Ramirez M. (2007). Resilience: A Concept Analysis. Nurs. Forum..

[11] Shilpa S, Srimathi NL. (2015). Role of Resilience on Perceived Stress among Pre University and Under Graduate Students. Int. J. Indian Psychol..

[12] Thomée S, Härenstam A, Hagberg M. (2011). Mobile phone use and stress, sleep disturbances, and symptoms of depression among young adults - a prospective cohort study. BMC Public Health..

[13] Pundir P, Andrews T, Binu VS, Kamath R. (2016). Association of problematic mobile phone use with psychological distress and self-esteem among college students in South India: a cross-sectional study. ijcmph.

[14] Solomon O. (2013). Exploring the relationship between resilience, perceived stress and academic achievement.

[15] Cohen S. (1983). Perceived Stress Scale..

[16] Connor KM, Davidson JRT. (2003). Development of a new Resilience scale: The Connor-Davidson Resilience scale (CD-RISC). Depress Anxiety..

[17] Lamond AJ, Depp C, Ph D, Allison M, Reichstadt J, Moore DJ (2009). Measurement and Predictors of Resilience Among Community- Dwelling Older Women. J. Psychiatr. Res..

[18] Walsh SP, White KM, Young RM. (2010). Needing to connect: The effect of self and others on young people's involvement with their mobile phones. Aust. J. Psychol..

[19] Innes SI. (2017). The relationship between levels of resilience and coping styles in chiropractic students and perceived levels of stress and well-being. J. Chiropr. Educ..

[20] Singh A, Chopra M, Adiba S, Mithra P, Bhardwaj A, Arya R (2013). A descriptive study of perceived stress among the North Indian nursing undergraduate students. Iran J. Nurs. Midwifery Res..

[21] Davydov D, Stewart R, Ritchie K, Chaudieu I. (2010). Resilience and mental health. Clin. Psychol. Rev..

